# Supplementary figures and images for: Silencing GhJUB1L1 (JUB1-like 1) reduces cotton (Gossypium hirsutum) drought tolerance
Source: PLoS One. 2021 Nov 5;16(11):e0259382. doi: 10.1371/journal.pone.0259382 (PMC8570493; doi:10.1371/journal.pone.0259382)

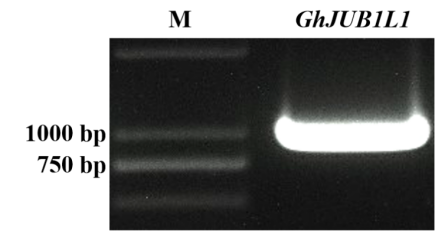

Supplement: S1 Fig — M, Marker DL2000. (TIF) [file pone.0259382.s001.tif]

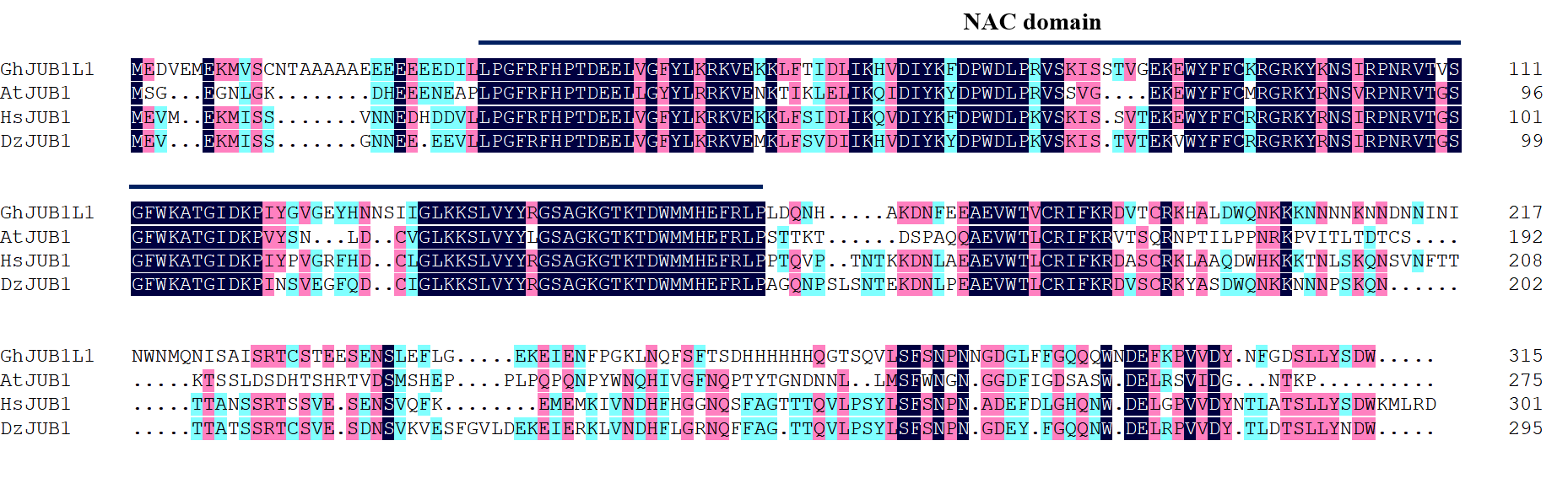

Supplement: S2 Fig — Horizontal lines, mazarine shading, and pink shading represent conserved NAC binding domains, conserved amino acid residues, and similar amino acid residues, respectively. (TIF) [file pone.0259382.s002.tif]

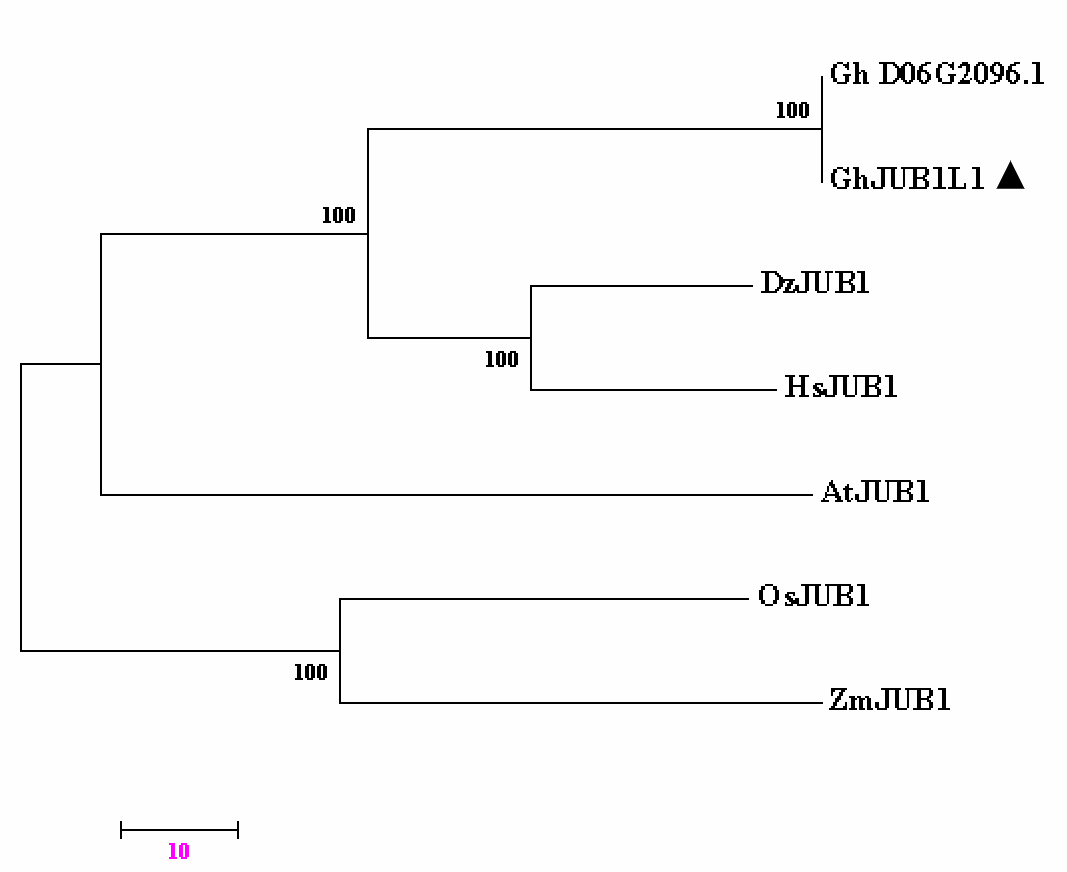

Supplement: S3 Fig — ▲ represents GhJUB1L1. (TIF) [file pone.0259382.s003.tif]

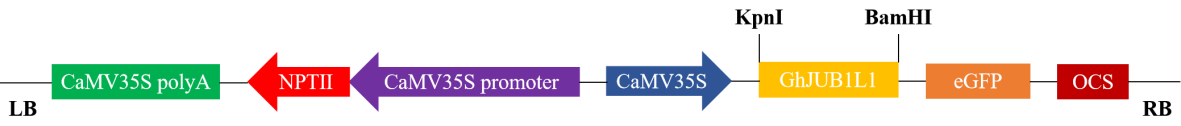

Supplement: S4 Fig — CaMV35S, promoter. (TIF) [file pone.0259382.s004.tif]

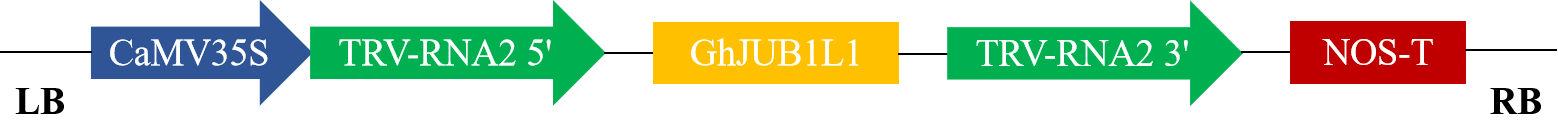

Supplement: S5 Fig — CaMV35S, promoter. (TIF) [file pone.0259382.s005.tif]

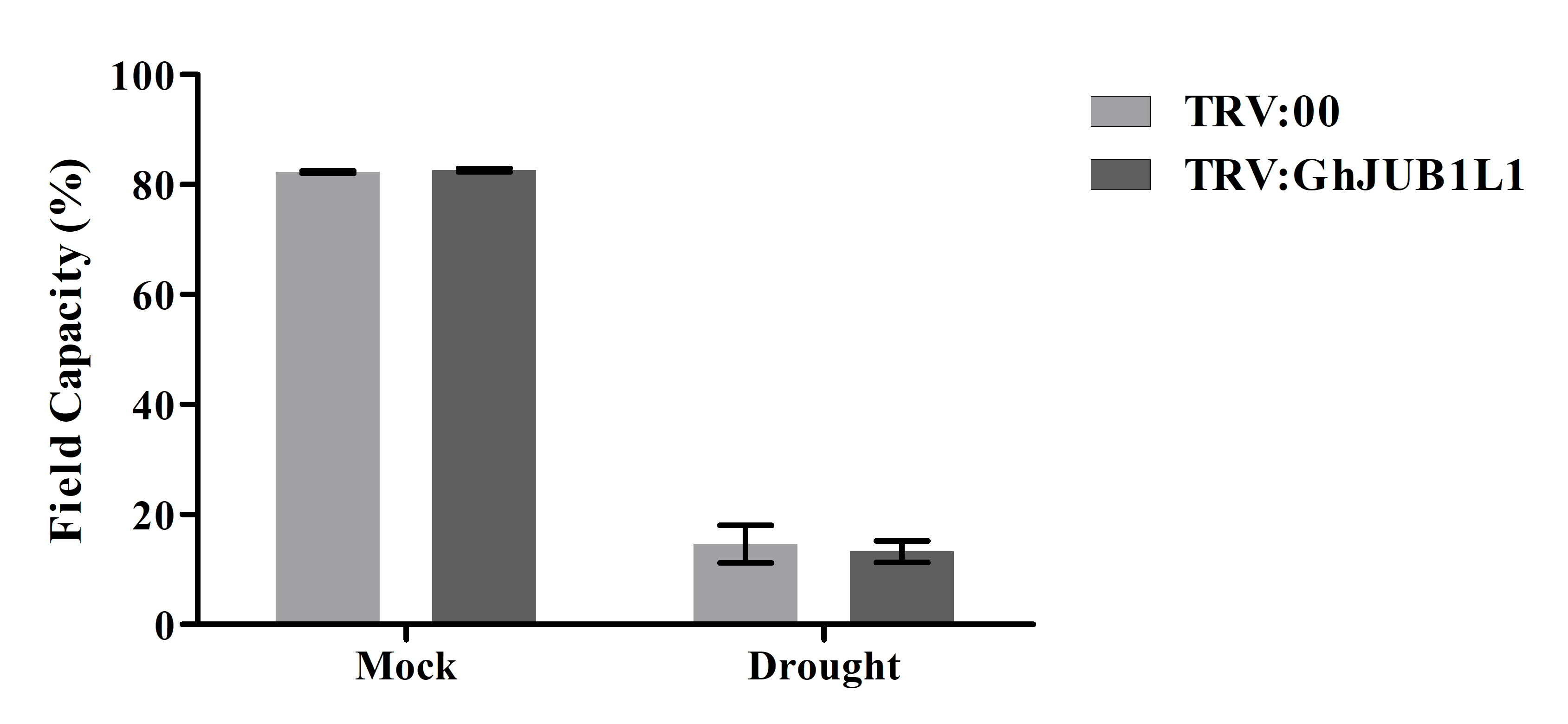

Supplement: S6 Fig — Error bars represent SD (standard deviation) of three independent replicates. (TIF) [file pone.0259382.s006.tif]

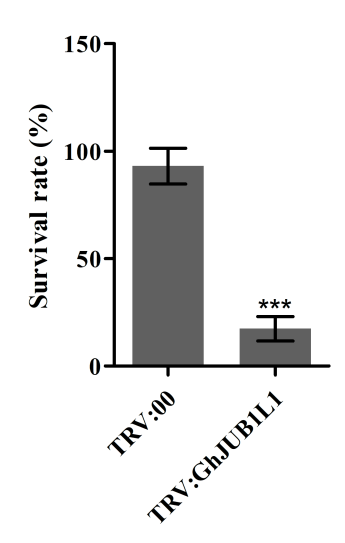

Supplement: S7 Fig — Error bars represent SD of three independent replicates. *** represent p<0.001. (TIF) [file pone.0259382.s007.tif]

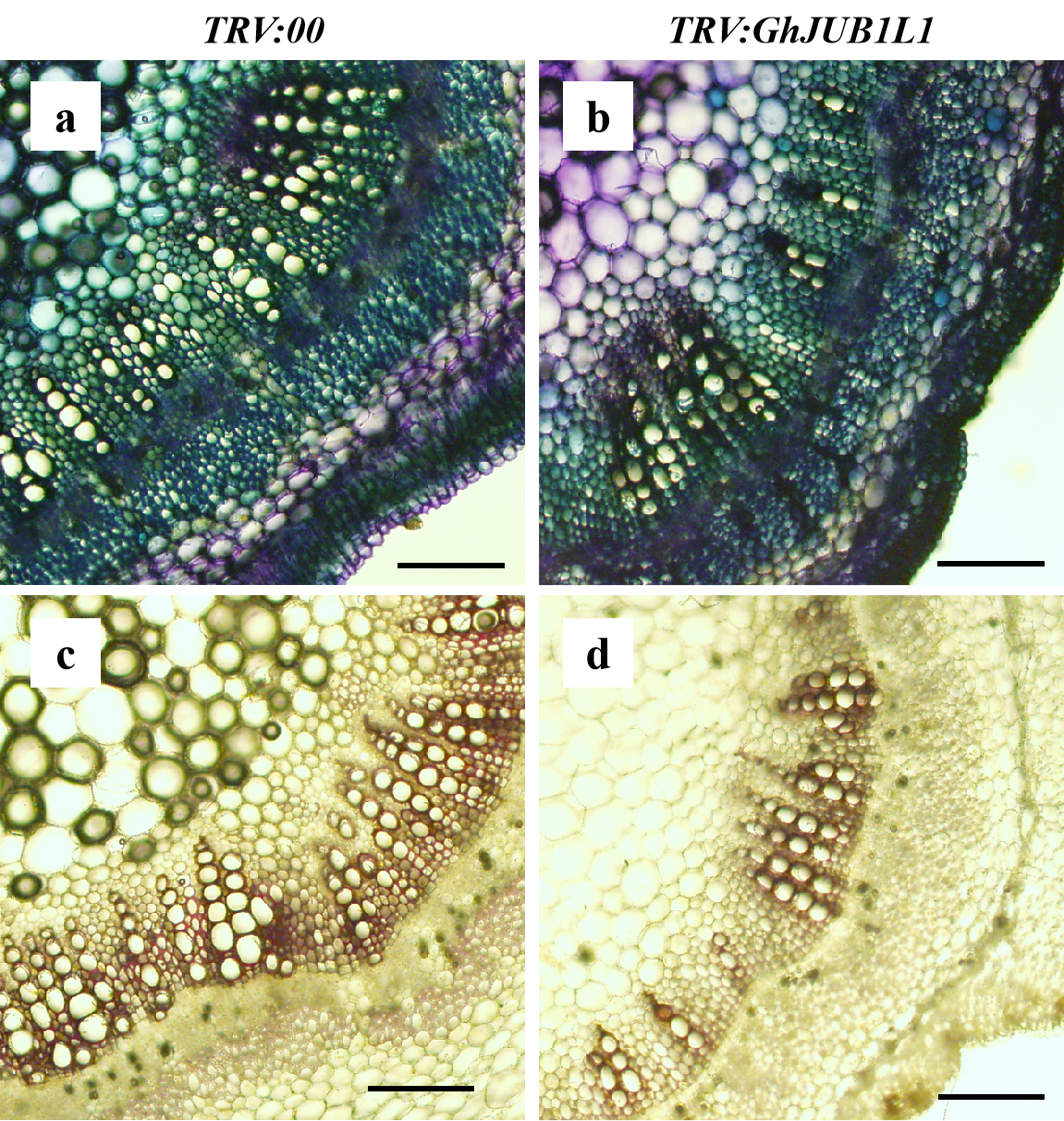

Supplement: S8 Fig — Phloroglucinol/HCl (a-b) and toluidine bluestaining (c-d) analysis for re-watered plants. Scale bar = 200 μm. (TIF) [file pone.0259382.s008.tif]
