# Supplementary material for: Silencing GhJUB1L1 (JUB1-like 1) reduces cotton (Gossypium hirsutum) drought tolerance
Source: PLoS One. 2021 Nov 5;16(11):e0259382. doi: 10.1371/journal.pone.0259382 (PMC8570493; doi:10.1371/journal.pone.0259382)
Supplement: S1 Table — (PDF) [file pone.0259382.s009.pdf]

Supplementary Information

S1 Table. Gene-specific primers used in isolation of *GhJUB1L1* genes and vector construction

Primer name	Primer sequence (5'-3')
JUB1L1-F	ATGGAAGATGTGGAGATGGAG
JUB1L1-R	CTACCAATCACTATAAAGCAATGAATC
TRV-JUB1L1-F	ttggggtaccgagctcgcccCAGCAATTTCAAGAACATGCAG
TRV-JUB1L1-R	aatgtcttcgggacatgcccCTGGCTTGAACTCATCGTTC
pGBKT7-JUB1L1-F	tggccatggaggccgaattcATGGAAGATGTGGAGATGGA
pGBKT7-JUB1L1-R	cgctgcaggtcgacggatccCCAATCACTATAAAGCAATG
pGBKT7-JUB1L1-N-R	cgctgcaggtcgacggatccTGGGAGACGAAATTCATGCATC
pGBKT7-JUB1L1-C-F	tggccatggaggccgaattcCTTGATCAAAATCACGCCAAAG
P2300-JUB1L1-eGFP-F	cggggtaccATGGAAGATGTGGAGATGGAG
P2300-JUB1L1-eGFP-R	cgcggatccCCAATCACTATAAAGCAATGAATCACC
0800-SOS2-F	atatcgaattcctgcagcccgtagagtaaactacacccatgg
0800-SOS2-R	ctagaactagtggatcccccgagagcttactctccatgctg
0800-ABI1-F	atatcgaattcctgcagcccagattgatgatgtggccatc
0800-ABI1-R	ctagaactagtggatcccccggacatgagaaggagaacgag

0800-CesA7-F	atatcgaattcctgcagcccgagccttccacttgtaaggtg
0800-CesA7-R	ctagaactagtggatccccctggaggatatgaaaggaggg
0800-CCoAOMT1-F	atatcgaattcctgcagcccccgctcatggatccttgtatg
0800-CCoAOMT1-R	ctagaactagtggatcccccggaactttgaattggtctgcc
0800-IRX14-F	atatcgaattcctgcagcccgcaatagcataacgtcgccg
0800-IRX14-R	ctagaactagtggatcccccgcgtggtttaggggaatttag
