# Supplementary material for: Silencing GhJUB1L1 (JUB1-like 1) reduces cotton (Gossypium hirsutum) drought tolerance
Source: PLoS One. 2021 Nov 5;16(11):e0259382. doi: 10.1371/journal.pone.0259382 (PMC8570493; doi:10.1371/journal.pone.0259382)
Supplement: S2 Table — (PDF) [file pone.0259382.s010.pdf]

S2 Table. Gene-specific primers used in RT-PCR analysis

Primer name	Primer sequence (5’-3’)
JUB1L1-RT-F	CCCATATGTATTCAGTCAGAGAC
JUB1L1-RT-R	TCTTCTTCTTCTGCTGCTGC
GhCesA4-RT-F	CTCTCCTTGAGGTTCGAGCGTG
GhCesA4-RT-R	ACAACACACTTTCTCGACCGGG
GhCesA7-RT-F	GGTTGCTGCCCTTGTTTTGGAC
GhCesA7-RT-R	TCGCTTCCCCATTCGGTTTTGT
GhCesA8-RT-F	CATCCTTGCCTTGGACTACCCG
GhCesA8-RT-R	ATTCAAATGTCAGCATGGCCGC
Gh4CL-RT-F	CGACGTCGTAGCGCTTCCTTAT
Gh4CL-RT-R	CTCGTGACCAAACCTTTGTGCG
GhCCOAOMT1-RT-F	GCTGTTGGGTCTGGGTTAGCTT
GhCCOAOMT1-RT-R	CTTGCTTTGGCTTCTGCAACGG
GhIRX9-F	GGACCTGTTTGCGATTCGTCAC
GhIRX9-R	TTCTGTGAAGTGCCTTGGACCG
GhIRX14-F	CACCAGAGCTACATCAGCCGTC

GhIRX14-R	GGATCGGAACGGCGACGTATAG
GhHis-F	CCGTCCTGGAACTGTTGCTCT
GhHis-R	ACCCACAAGGTATGCCTCTGC
