# Supplementary material for: Silencing GhJUB1L1 (JUB1-like 1) reduces cotton (Gossypium hirsutum) drought tolerance
Source: PLoS One. 2021 Nov 5;16(11):e0259382. doi: 10.1371/journal.pone.0259382 (PMC8570493; doi:10.1371/journal.pone.0259382)
Supplement: Attachment — Submitted filename: Response to Reviewers.doc [file pone.0259382.s011.doc]

**Journal Requirements:**

When submitting your revision, we need you to address these additional requirements.

1. Please ensure that your manuscript meets PLOS ONE's style requirements, including those for file naming.

**Answer:** Thank you for your careful comment. We have carefully checked our manuscript.

2. We suggest you thoroughly copyedit your manuscript for language usage, spelling, and grammar. If you do not know anyone who can help you do this, you may wish to consider employing a professional scientific editing service.

**Answer:** Thank you for your careful comment. We have carefully checked our manuscript.

**Reviewer 1**

Major comments:

In material and methods section: Please add reference for the protocols used for staining of cellulose and others. NO clarity of sample collection for qRT, staining etc. Mention after how many days of silencing symptoms (albino) the samples were collected for qRT, staining. After how many days of re-watering the phenotype was recorded. Drought stress imposition protocol is missing-after how many days of silencing the drought stress was imposed? Mention the % field capacity after 10days of withholding water.

**Answer:** Thank you for your careful comment. We have given detailed protocol in the Methods section. And the % field capacity after 10 days of withholding water was given in S6 Fig.

Results section:

1. Need to provide physiological data eg. RWC or EC etc., to support that plant are experiencing stress and control plants are unstressed.

**Answer:** Accepted and added.

2. In Fig4 and other expression data-normalization against what?

**Answer:** Thank you for your advice. The expression level of GhJUB1L1 gene in samples from root, mock, and TRV:2096-3 was set as 1 to figure 2a, 2b, and 5b, and then the expression levels of GhJUB1L1 gene in other samples was normalized to these for figure 2a, 2b, and 5b, respectively. The expression level of these genes in samples from TRV:00 was set as 1 to figure 7, and then the expression levels of these gene in other samples was normalized to these for figure 7, respectively.

3. Fig 4b- what is drought 1, 2 and 3?

**Answer:** Thank you for your careful comment. We have corrected.

4. What statistical parameter (ttest, ANOVA) was used is not mentioned.

**Answer:** Thank you for your good suggestion. We used t-test for statistical analysis and have added in the Methods section.

5. It would be nice to have the staining of re-watered plants.

**Answer:** Thank you for your valuable suggestions. We have added the staining images in S8 Fig.

6. Gene name must be italicized throughout. It is better to use drought tolerance rather than drought resistance.

**Answer:** Thank you for your advice. We have carefully checked our manuscript and used italics for genes, and replaced drought resistance to drought tolerance.

7. No discussion about why of the two genes expressed (heat map) in stem only one was chosen for study-criteria used?

**Answer:** Thank you for your valuable suggestions. We have explained it in the Results section.

In general, the language can be improved as it is not clear is most places. The sentences can be reframed to make it simple and clear.

**Answer:** Thank you for your valuable advices. We have carefully checked our manuscript.

8. Line 44-46-not clear

**Answer:** Thank you for your advice. We have corrected.

9. Line 56- remove and after wheat

**Answer:** Accepted and corrected.

10. Line 58- reframe as-which plays role in regulating longevity and stress tolerance

**Answer:** Accepted and corrected.

11. Line 70-reframe as-In addition, ectopic expression of AtJUB1 led to enhanced stress tolerance and reduced oxidative damage.

**Answer:** Accepted and corrected.

12. Line 73- growth and stress response interactor

**Answer:** Accepted and corrected.

13. Line 76- drought induced gene

**Answer:** Accepted and corrected.

14. Line 128- to silence

**Answer:** Accepted and corrected.

15. Line 130-remove and then,

**Answer:** Accepted and corrected.

16. Line 146-reframe- once the material turns red, cover the glass quickly and observe under a normal microscope.

**Answer:** Accepted and corrected.

17. Line 149- accorded to the intensity of pigmentation

**Answer:** Accepted and corrected.

18. Line 150-it is browning

**Answer:** Accepted and corrected.

19. Line 165-predominantly not predominance

**Answer:** Accepted and corrected.

20. Line 168-Based on above analysis, Gh…..was selected for further study which was cloned and renamed as GhJUB1L1

**Answer:** Accepted and corrected.

21. Line 209- the results indicated tthat expression level of GhJUB1L1 was higher in stem and leaf while it was lower in flowers and roots under non-stress conditions. The expression of this gene in these tissues increased when plants were subjected to drought.

**Answer:** Accepted and corrected.

22. Line 217- in/different

**Answer:** Accepted and corrected.

23. Line 225- in the nucleus

**Answer:** Accepted and corrected.

24. Line 276- not clear

**Answer:** Accepted and corrected.

25. Line 281, line 288-289- reframe

**Answer:** Accepted and corrected.

26. Line 353- a homolog instead of one homolog

**Answer:** Accepted and corrected.

**Reviewer 2:**

Overall the design of the study is good and authors wrote conveyed the research findings very well. I have few suggestions for authors to improve the manuscript quality.

1. Please change all the figure legends (figure 1, 2, 3, 5, 6, 8 and 9). They were very hard to read during the review process. Also maintain same size and font for figure legends.

**Answer:** Thank you for your valuable suggestions. We have corrected.

2. Other than qRT-PCR data of downstream targets. authors have not shown any direct evidence like yeast one hybrid assay or luciferase reporter assay with few downstream target gene promoters. It would add more strength to the research findings.

**Answer:** Thank you for your valuable suggestions. We have added luciferase reporter assay with five downstream target gene promoters in Fig 8.

3. Please rename the GhCCOAOMT1 to GhCCoAOMT1 across the manuscript.

**Answer:** Accepted and corrected.
